# Effect of Mechanical Environment Alterations in 3D Stem Cell Culture on the Therapeutic Potential of Extracellular Vesicles

**DOI:** 10.34133/bmr.0189

**Published:** 2025-05-23

**Authors:** Wu Young Kang, Sunyoung Jung, Hyundoo Jeong, Hyun-Myung Woo, Min-Ho Kang, Hojae Bae, Jae Min Cha

**Affiliations:** ^1^ Department of Biomedical & Robotics Engineering, College of Engineering, Incheon National University, Incheon 22012, Republic of Korea.; ^2^ 3D Stem Cell Bioengineering Laboratory, Research Institute for Engineering and Technology, Incheon National University, Incheon 22012, Republic of Korea.; ^3^ Department of BioMedical Sciences, Seoul National University College of Medicine, Seoul 03080, Republic of Korea.; ^4^ Department of BioMedical-Chemical Engineering (BMCE), The Catholic University of Korea, Bucheon 14662, Republic of Korea.; ^5^ Department of Biotechnology, The Catholic University of Korea, Bucheon 14662, Republic of Korea.; ^6^ Department of Stem Cell and Regenerative Biotechnology, KU Convergence Science and Technology Institute, Konkuk University, Seoul 05029, Republic of Korea.; ^7^ Institute of Advanced Regenerative Science, Konkuk University, Seoul 05029, Republic of Korea.

## Abstract

Stem-cell-derived extracellular vesicles (EVs) have emerged as a promising therapeutic option, addressing the limitations of conventional stem cell therapies. However, the variability and poorly defined therapeutic contents of EVs produced under standard 2-dimensional culture conditions present challenges for their clinical application. In this study, we investigated how the therapeutic properties of mesenchymal stem cell (MSC)-derived EVs can be enhanced by culturing MSCs within 3-dimensional hydrogels that have tunable mechanical properties. Our results demonstrate that different mechanical cues from the culture environment can induce specific gene expression changes in MSCs without compromising their inherent characteristics. Furthermore, EVs derived from these MSCs exhibited distinct angiogenic and immunomodulatory activities, which were dependent on the mechanical properties of the hydrogels used. A comprehensive analysis of the cytokines and microRNAs present in the EVs provided additional validation of these findings. By utilizing a noninvasive culture method that eliminates the need for genetic modification or exogenous biochemical supplementation, our approach presents a novel platform for the tailored production of EVs, thereby enhancing their therapeutic potential in regenerative medicine.

## Introduction

Extracellular vesicles (EVs) facilitate intercellular communication by transferring multiple signaling factors, including cytokines, growth factors, and nucleic acids, while enclosed in lipid membranes that share transmembrane molecules with their parent cells [1]. EVs derived from mesenchymal stem cells (MSCs) engage in the multifactorial therapeutic effects of MSCs, such as angiogenesis, tissue regeneration, and immunomodulation [2]. Therapies using MSC-EVs, which demonstrate a better safety profile and lower immunogenicity than MSC transplantation therapies, could circumvent existing limitations in cell-based treatments, such as pulmonary embolism, ectopic tissue formation, immune rejection, and tumorigenesis [2–4]. In addition, as a therapeutic agent, EVs offer logistical advantages for large-scale manufacturing, storage, and shipping compared to their parent cells, thus extending the range of clinical application strategies for patient administration [5]. Therefore, the therapeutic applications of MSC-EVs targeting various intractable diseases are at the forefront of clinical investigations.

Despite their potential for application to regenerative therapies, MSC-EVs are limited in their clinical uses owing to the indistinct therapeutic contents obtained by routine 2-dimensional (2D) culture conditions [6]. Thus, it is crucial to regulate the wide-ranging therapeutic contents of MSC-EVs to maximize their therapeutic efficacy for targeted disease treatment. Many studies have attempted to induce changes in the therapeutic contents of EVs through gene transfection or additional biochemical supplementation during parent cell culture [7]. However, these methods often directly alter the parent cell characteristics, involve high costs, and introduce complexities into the stability assessment of genetic and/or chemical supplementary combinations [8]. Overcoming these challenges requires the development of advanced culture techniques that can offer the sustained and controlled modulation of parent cell characteristics, thus leading to their successful integration into the therapeutic capacities of the EVs [9].

Cells modulate their behavior by responding to their 3-dimensional (3D) physical microenvironment, which consists of the extracellular matrix (ECM) that they produce themselves [10]. In recent years, hydrogels, characterized by a network of hydrophilic porous polymers capable of retaining substantial amounts of water, have been regularly used as effective 3D cell culture platforms [11,12]; 3D cell cultures using hydrogels with modulation of mechanical properties could provide cells with controlled physical stimuli to alter their characteristics, thereby possibly allowing the manipulation of cargo sorting in EVs without external chemical interventions [11]. Gelatin methacrylate GelMA) hydrogels can be covalently cross-linked via the radical photopolymerization of methacryloyl substituents incorporated into gelatin in the presence of a photoinitiator and exposure to ultraviolet (UV) light [13]. GelMA hydrogels have emerged as promising biomaterials because, as a 3D cell culture matrix, they can provide the advantages of both the arginine–glycine–aspartic acid sequence and metalloproteinase-responsive peptide motifs. They also display reproducible and tunable physicochemical properties while being biocompatible, biodegradable, noncytotoxic, and nonimmunogenic [14].

**Fig. 1.**
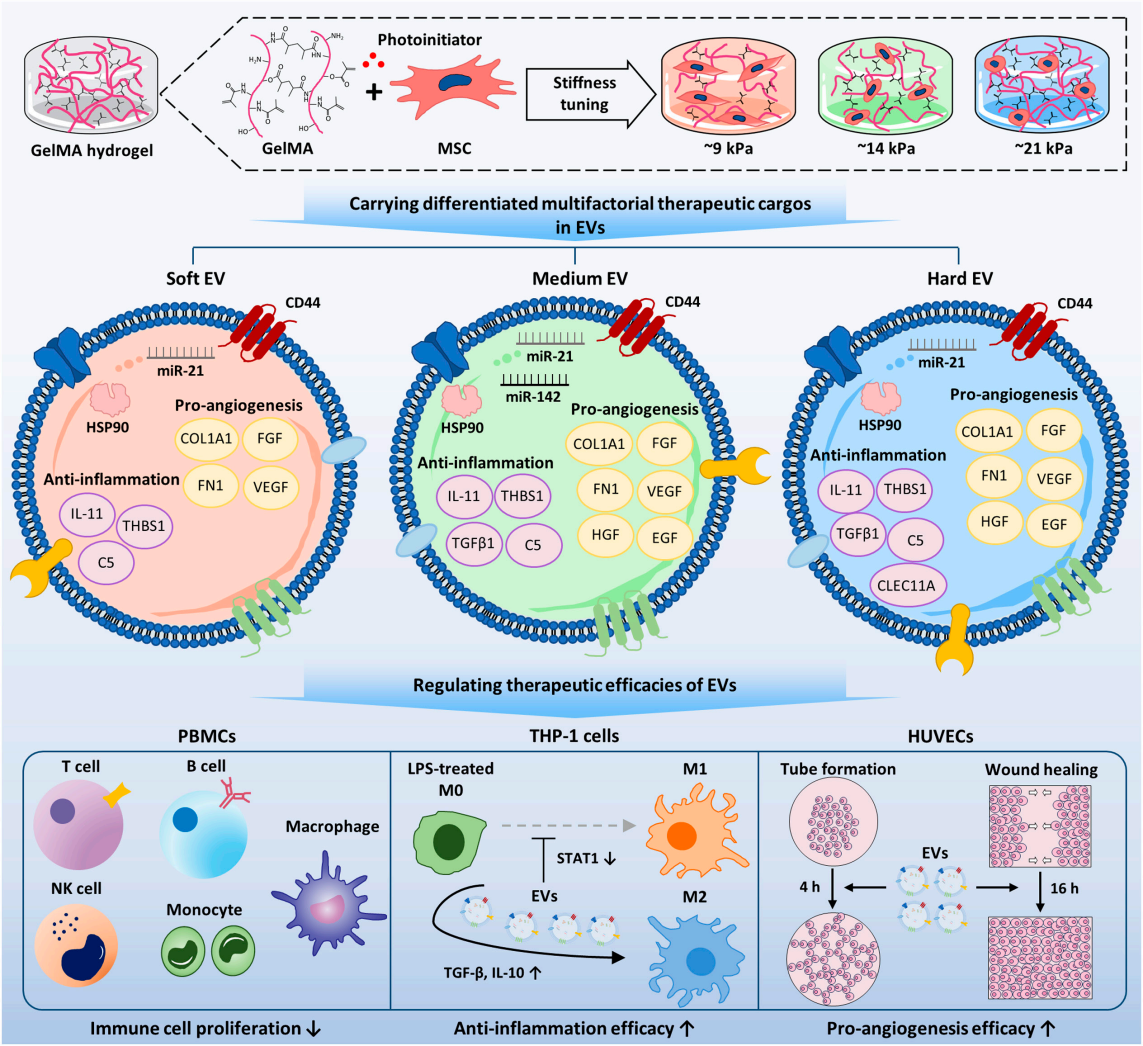
Schematic diagram of the regulation of the differentiated multifactorial therapeutic cargo of extracellular vesicles (EVs) and their efficacies through mechanical microenvironment alteration. GelMA, gelatin methacrylate; MSC, mesenchymal stem cell; HSP90, heat shock protein 90; IL-11, interleukin 11; THBS1, thrombospondin-1; COL1A1, collagen type I alpha 1; FN1, fibronectin 1; FGF, fibroblast growth factor; VEGF, vascular endothelial growth factor; TGFβ1, transforming growth factor beta 1; HGF, hepatocyte growth factor; EGF, epidermal growth factor; CLEC11A, C-type lectin domain family 11-member A; PBMCs, peripheral blood mononuclear cells; NK, natural killer; LPS, lipopolysaccharide; STAT1, signal transducer and activator of transcription 1; HUVECs, human umbilical vein endothelial cells.

In this study, we aimed to modulate the angiogenic and immunomodulatory capacities of EVs by culturing MSCs encapsulated in GelMA hydrogels with controlled mechanical properties. Our in vitro efficacy testing models, related to immunomodulation, angiogenesis, and wound-healing processes, demonstrated the differentiated therapeutic capacities of EVs according to their culture matrix properties. We systematically characterized and examined the resulting EVs and their cargo compounds, including cytokines and microRNAs (miRs). In addition, comprehensive proteomic analysis of EVs using liquid chromatography–mass spectrometry (LC–MS) and bioinformatics further supported our results (Fig. 1). Our study demonstrates the possibility of regulating the therapeutic capacity of MSC-EVs by providing MSCs with different mechanical microenvironments, rather than using genetic modification or biochemical factor supplementation during culture.

## Materials and Methods

### Cell culture

Low-glucose Dulbecco’s modified Eagle’s medium (DMEM; Life Technologies Corporation, Carlsbad, CA, USA) with 10% fetal bovine serum (FBS; Life Technologies Corporation) and 1% antibiotic-antimycotics (Life Technologies Corporation) was used for the expansion of human-bone-marrow-derived MSCs, a single lot of which was continuously used throughout this study (Lonza, Basel, Switzerland). MSCs were cultured in 100-mm culture dishes (Corning, Tewksbury, MA, USA) and passaged up to P6 prior to seeding for EV harvest. Encapsulated and 2D-cultured MSCs were cultured in DMEM supplemented with 10% exosome-free FBS (System Biosciences, Palo Alto, CA, USA) and 1% antibiotic-antimycotics. Human umbilical vein endothelial cells (HUVECs; PromoCell, Heidelberg, Germany) were cultured in medium 199 (Lonza) with 20% FBS, 1% antibiotic-antimycotics, 3 ng/ml recombinant human fibroblast growth factor-basic (bFGF; Life Technologies Corporation), and 500 U of heparin (Sigma-Aldrich, St. Louis, MO, USA). Human peripheral blood mononuclear cells (PBMCs; STEMCELL Technology, Vancouver, Canada) were cultured in RPMI 1640 (Life Technologies Corporation) supplemented with 10% FBS and 1% antibiotic-antimycotics. Human monocytic cell lines derived from an acute monocytic leukemia patient (THP-1; American Type Culture Collection, Manassas, VA, USA) were cultured in RPMI 1640 with 10% FBS, 1% antibiotic-antimycotics, and 0.05 mM 2-mercaptoethanol (Life Technologies Corporation). All cells were cultured in an incubator at 37 °C in a humidified atmosphere containing 5% CO_2_.

### GelMA synthesis

Porcine gelatin (Sigma-Aldrich) was dissolved in 10% (w/v) phosphate-buffered saline (PBS; Life Technologies Corporation) by double-boiling. Methacrylic anhydride (Sigma-Aldrich), serving as a cross-linking agent, was slowly added to the gelatin solution at 50 °C under continuous magnetic stirring, at a volume equivalent to 20% of PBS. To ensure effective methacrylation, special attention was given to preventing abrupt pH fluctuations caused by the addition of methacrylic anhydride. The reagent was added at a rate of 0.5 ml/min, and whenever the pH dropped below 5.5, the addition was carefully controlled to maintain the pH at 5.5 or above. After 2 h, the reaction was terminated by reducing the temperature to 40 °C and diluting the starting gelatin solution 4-fold with PBS. The solution was dialyzed against fresh distilled water through Spectra dialysis tubing with 12- to 14-kDa molecular weight cutoffs (Repligen, Waltham, MA, USA) for a week, followed by lyophilization.

### Cell encapsulation into GelMA hydrogel

Glass slides were washed with 10% (w/v) sodium hydroxide (Duksan General Science, Seoul, Korea) and distilled water to remove impurities. The washed slides were treated with a solution of 3-trimethoxysilyl propyl methacrylate (TMS-PMA; Sigma-Aldrich) and a polyethylene glycol dimethacrylate (PEG; Polysciences, Inc., Warrington, PA, USA) solution containing 20% PEG and 0.05% (w/v) 2-hydroxy-4′-(2-hydroxyethoxy)-2-methylpropiophenone (Irgacure 2959; Sigma-Aldrich), as a photoinitiator, in PBS to prevent nonspecific cell absorption. Before encapsulation, polydimethylsiloxane (SYLGARD 184 Silicone Elastomer, Dow Corning, Midland, MI, USA) spacers with a height of 300 μm were fabricated for the encapsulation process. For MSC encapsulation, GelMA hydrogel solution containing 10% (w/v) GelMA and 0.05% (w/v) Irgacure 2959 in PBS was freshly prepared and used immediately. MSC pellets were suspended in GelMA solution and medium at a ratio of 5:1 and a density of 1.5 × 10^5^ cells, resulting in a final GelMA concentration of 8.3%. Subsequently, 75 μl of GelMA solution containing suspended MSCs was prepared on a TMS-PMA- and PEG-precoated slide glass. MSCs were encapsulated within cross-linked GelMA with differently regulated mechanical stiffnesses, namely, soft (3.8 mW/cm^2^ for 3 s), medium (3.8 mW/cm^2^ for 10 s), and hard (3.8 mW/cm^2^ for 20 s), using UV radiation (OmniCure S2000, Excelitas Technologies Corp., Waltham, MA, USA). The encapsulated MSCs were subsequently cultured for 5 d with 3D orbital shaking at 20 rpm (FINEPCR, Gunpo, Korea). The culture medium was not changed during the culture period.

### Atomic force microscopy measurement

The mechanical properties of the GelMA hydrogels were evaluated on a force–distance curve using an atomic force microscope (Park Systems, Suwon, South Korea) equipped with a liquid probe hand. Force–distance curve measurements were performed in a liquid environment filled with PBS solution, with data recorded at 5 randomly selected points for each sample and averaged. A pyramidal silicon nitride probe (NanoWorld, Neuchâtel, Switzerland) with a nominal spring constant of 0.08 N m^−1^ was used for the measurements. The Young’s moduli of the hydrogels were calculated from the approaching parts of the recorded individual force curves using the Hertzian model.

### Focused ion beam quanta 3D imaging of GelMA hydrogel

A Quanta 3D field emission gun (FEI, Hillsboro, OR, USA) with an Alto 2500 cryo-transfer system (Gatan, Pleasanton, CA, USA) was used to perform cryo-focused ion beam (FIB) Quanta 3D imaging of the GelMA hydrogel. The hydrogels on the PEG-coated glass slides were affixed to a copper stub using carbon tape. A liquid nitrogen slush was induced by evacuating the freezing chamber. The samples were then submerged in the chamber and directly transferred to a cold stage. Samples were etched at −90 °C for 3 min to remove the ice formed during rapid freezing. The sample substrates were coated with Au/Pd to prevent charging. Cryo-scanning electron microscopy images were acquired within the FIB chamber.

### Encapsulated MSC viability and proliferation assessments

Live/dead assays were conducted using the LIVE/DEAD Viability/Cytotoxicity Kit (Life Technologies Corporation) to examine the viability of the encapsulated MSCs after 5 d of culture. Briefly, the LIVE/DEAD solution, consisting of a calcein AM/ethidium homodimer ratio suggested by the manufacturer, was applied to the samples, which were then incubated for 30 min at room temperature (RT) and washed with PBS. LIVE/DEAD (calcein AM/ethidium homodimer)-labeled cells were visualized using a confocal microscope (Leica Microsystems, Wetzlar, Germany). A DNA quantification assay (Life Technologies Corporation) was performed to quantify the number of encapsulated MSCs after 5 d of culture, following the manufacturer’s instructions. Before assessment, collagenase A (Sigma-Aldrich) was used for GelMA hydrogel degradation.

### Immunocytochemistry

MSCs were fixed in 4% (w/v) paraformaldehyde (Biosesang, Seongnam, South Korea) for 10 min at 4 °C and permeabilized in 0.2% Triton X-100 (SAMCHUN, Seoul, Korea) for 15 min. Each sample was blocked in 4% bovine serum albumin (BSA; Sigma-Aldrich) and Alexa Fluor 488 phalloidin (Life Technologies Corporation) to visualize the cytoskeletal structure of the cells. To characterize the MSCs, 2D control MSCs were incubated with CD105 primary antibody (Abcam, Cambridge, UK) and Alexa Fluor 488 anti-mouse immunoglobulin G (IgG) secondary antibody (Abcam), both at a 1:1,000 dilution. The samples were incubated with 4′,6-diamidino-2-phenylindole (Life Technologies Corporation) in a dark room. Throughout the process, PBS washing was performed. The samples were preserved using a mountant (Life Technologies Corporation), and images were captured using confocal microscopy.

### Quantitative real-time polymerase chain reaction reverse transcription analysis

On day 5 of the culture, total RNA was extracted from the encapsulated MSCs. Collagenase A (Sigma-Aldrich) was used for GelMA hydrogel degradation. Total RNA was extracted using TRIzol reagent (Life Technologies Corporation) for subsequent gene expression analysis. Complementary DNA was prepared by reverse transcription using ReverTra Ace qPCR-RT Master Mix (TOYOBO, Osaka, Japan). Quantitative real-time polymerase chain reaction reverse transcription (qRT-PCR) was carried out using RT2 Profiler PCR arrays (Qiagen, Hilden, Germany). The RNA secreted by each hydrogel group was compared with that from the control group, and the results are presented as a heat map. Subsequently, the RNA results were categorized by pathway, and the pathways with the most marked differences compared to the control group were highlighted. The RNAs that were highly secreted in each group compared to the control group were selected and listed using the Gene Ontology (GO) platform and annotated (http://bioinformatics.sdstate.edu/go/).

Total miR was extracted from the EVs using the miRNeasy Serum/Plasma Kit (Qiagen). Complementary DNA was prepared by reverse transcription using the TaqMan Micro-RNA Reverse Transcription Kit (Life Technologies Corporation). qRT-PCR was carried out using TaqMan Fast Advanced Master Mix, and all methods using kits followed the manufacturer’s instructions.

### Isolation of EVs from the encapsulated MSC culture medium

Encapsulated MSC-conditioned media were collected on day 5 of culture. Following the removal of the media from each well, 4 ml of PBS was added to every 3 wells and gently shaken before harvesting any residual EVs. The harvested media were then ultracentrifuged at 2,500 × g for 10 min at RT, and the supernatant was filtered using a 0.22-μm polyethersulfone membrane filter bottle (Corning) to eliminate cell debris. Subsequently, the filtered media were loaded into a tangential flow filtration (TFF) system (Cole Parmer, Vernon Hills, IL, USA) with an Omega 300-kDa membrane (Pall, New York, NY, USA). In the TFF process, a concentration of 190 ml was used, and diafiltration with 10 ml of PBS was repeated 10 times using 200 ml of medium. This resulted in a final concentration of 8.5 ml for each sample. If characterization of the EV pellet was required, the TFF-concentrated sample was ultracentrifuged at 100,000 × g for 1 h at 4 °C, and the supernatant was discarded. The collected EV pellets were washed with PBS.

### Characterization of EVs by cryogenic transmission electron microscopy and tunable resistive pulse sensing with detergent treatment

For morphological characterization of the EVs, the EVs (3 or 4 μl) were added on a grid (Quantifoil, R 1.2/1.3, 200 mesh, EMS) and blotted for 1.5 s at 4 °C or 4 s at 15 °C at 100% humidity. The EVs were plunge-frozen using Vitrobot Mark IV (FEI Company, Hillsboro, Oregon, USA) in liquid ethane and investigated using Talos L120C (FEI Company) at 120 kV at the Nanobioimaging Center (Seoul National University, Korea) or Glacios (Life Technologies).

The size distribution and concentration of the collected EVs were assessed using a tunable resistive pulse sensing (TRPS) system. Experiments were performed using qNano (Izon Science Ltd., Christchurch, New Zealand). Nanopores (NP200, Izon Science Ltd.) and calibration particles (CPC100, Izon Science Ltd.) were used to quantify the EVs. All reagents used for the qNano measurements were filtered through a 0.22-μm polyethersulfone membrane syringe filter (Sartorius, Göttingen, Germany) immediately before use. For the structural characterization of EVs that were enveloped by lipid membranes, 3% Triton X-100 was added to EVs derived from MSCs, and the EVs were lysed for 30 min. Subsequently, the size distribution of the EVs was analyzed using the TRPS system.

### Sodium dodecyl sulfate–polyacrylamide gel electrophoresis and western blot analysis

The MSC and EV pellets obtained after ultracentrifugation were resuspended in 5× radioimmunoprecipitation assay buffer (RIPA) containing a protease inhibitor cocktail (Roche, Basel, Switzerland), followed by lysis for 30 min and sonication. The samples were centrifuged at 13,200 rpm at 4 °C, and the supernatant was collected. The protein concentration was quantified using a bicinchoninic acid (BCA) protein assay (Invitrogen) according to the manufacturer’s protocol. The MSC and EV lysate (30 μg) was mixed with 4× sodium dodecyl sulfate sample buffer and heated at 95 °C for 5 min. Proteins were loaded onto 7.5% to 15% polyacrylamide gels and subjected to electrophoresis at 75 to 150 V for approximately 2 h using Tris–glycine running buffer. After electrophoresis, proteins were transferred using polyvinylidene fluoride membranes, followed by blocking for 45 min with 3% BSA at RT. Proteins were detected by incubation with primary antibodies (Alix [Santa Cruz Biotechnology, CA, USA, 1:1,000], TSG-101 [Santa Cruz Biotechnology, 1:1,000], CD63 [Santa Cruz Biotechnology, 1:1,000], and CD44 [Cell Signaling Technologies, Danvers, MA, USA, 1:1,000]) diluted in 1% BSA solution overnight at 4 °C. The membrane was washed 3 times for 5 min each with TBS containing 0.1% Triton X-100 (TBS-T) and then incubated with a goat anti-mouse IgG secondary antibody (Enzo Life Sciences, NY, USA, 1:10,000) in TBS-T for 1 h at RT. The incubated membrane was washed 5 times with TBS-T and visualized using a GeneGnome system (Syngene, Bangalore, India).

### Angiogenic efficacy test

For the tube-formation assay, a μ-slide 15-well plate (Ibidi, Martinsried, Germany) was coated with Matrigel (Corning) and incubated for 30 min at 37 °C prior to the experiment. HUVECs were trypsinized from the culture dishes and counted. The cell suspension was added to the upper well. For the wound-healing assay, HUVECs were pre-seeded on Culture-Insert 2-well plates in a 35-mm μ-slide (Ibidi) and incubated at 37 °C with 5% CO_2_ for at least 24 h. EVs isolated from encapsulated or 2D-cultured MSCs, and 3 μg/ml of vascular endothelial growth factor (VEGF) as a positive control, were used to treat each well at a concentration of 5 × 10^8^ particles/ml. The μ-slide was covered with the supplied lid and incubated at 37 °C with 5% CO_2_ for 4 or 16 h. Efficacy assays were performed using an optical microscope (Olympus, Tokyo, Japan). Quantitative differences in tube formation and migration were determined using the ImageJ software and normalized to the PBS-treated group.

### Immunomodulatory efficacy test

For the cell proliferation assay, PBMCs were seeded into a 96-well plate to quantify cellular proliferation. EVs isolated from encapsulated or 2D-cultured MSCs, along with immune stimulators including 10 μg/ml of lipopolysaccharide (LPS; Sigma-Aldrich) as a positive control, were used to treat each well at concentrations of 5 × 10^8^ particles/ml. After 72 h, the proliferation rate of PBMCs was determined using Cell Counting Kit-8 (Dojindo, Kumamoto, Kyushu, Japan) according to the manufacturer’s instructions. All experimental data were normalized to those of the PBS-treated group.

THP-1 cells were seeded into a 6-well plate to evaluate the immunomodulatory efficacy of EVs. THP-1 cells were differentiated into macrophage phenotype 0 (M0) cells by adding 5 ng/ml phorbol 12-myristate 13-acetate (Sigma-Aldrich). After 24 h, 10 μg/ml of LPS was added to each group of EVs at concentrations of 5 × 10^8^ particles/ml to induce differentiation into macrophage phenotype 1 (M1), and the cells were harvested after 2 d.

### Cytokine array analysis

The EVs were lysed using RIPA (Life Technologies Corporation) containing protease inhibitors (Roche). The protein content in the collected EVs was quantified using a Micro BCA Protein Assay Kit (Life Technologies Corporation) following the manufacturer’s instructions. For cytokine array analysis, lysates of the isolated EVs were applied to cytokine arrays (the human angiogenesis cytokine array and human inflammatory cytokine array; Abcam) according to the manufacturer’s instructions. Cytokine array membranes were developed using a chemiluminescence imager (GeneGnome XRQ, Syngene, Bangalore, India). Quantitative differences in cytokine expression were determined using the ImageJ software (National Institutes of Health, Bethesda, MD, USA). The intensities of the positive control signals were used to normalize the signal responses and compare the relative expression levels according to the manufacturer’s instructions.

### Proteomic analysis by LC–MS

EVs were lysed using ProEX CETi lysis buffer with inhibitors (Translab, Daejeon, Korea). EV proteins were measured using the Pierce BCA Protein Assay Kit (Life Technologies Corporation) and a microplate reader (TECAN, Männedorf, Switzerland) following the manufacturer’s instructions. Each 10 μg of protein from the MSC-EVs was digested in the solution of the Tryptic Digestion and Guanidination Kit (Life Technologies Corporation) for LC–MS. Briefly, the EV proteins of each group were denatured and reduced using ammonium bicarbonate and 1,4-dithiothreitol at 95 °C for 5 min. The samples were then alkylated in iodoacetamide in the dark at RT for 20 min, followed by tryptic digestion at 37 °C overnight. Guanidination reagent was added to the digested samples, which were incubated at 65 °C for 12 min. Finally, all peptide samples were loaded into Pierce C-18 spin columns (Life Technologies Corporation) for desalting, followed by lyophilization, and stored at −80 °C until LC–MS/MS analysis. Samples were recovered with solvent A (0.1% formic acid in deionized water) and analyzed using an UltiMate 3000 RSLCnano system connected to a Q Exactive Orbitrap Plus MS Spectrometer (Life Technologies Corporation) with Acclaim PepMap 100 and PepMap RSLC C18 columns.

### Bioinformatic analysis

Because there were relatively large differences in protein abundance levels between the different experimental groups, it was difficult to obtain the relative changing patterns in each experimental group using direct comparisons of their protein abundance levels. First, we verified the abundance levels of “angiogenesis” and “immune” proteins by accessing EBI QuickGO, which is a Web-based big data platform for GO and annotation (www.ebi.ac.uk/QuickGO/). Next, we extracted the proteins associated with the aforementioned GO terms and compared their abundance levels using log-transformed mean values. Then, to normalize the protein abundance level, we obtained *z* scores for each protein and visualized the protein abundance level for each experimental group using a heat map and balloon plots. Note that the *z* score is given by $z=\left(x-\mu \right)/\sigma$, where *x* is the protein abundance level and *μ* and *σ* are the mean and standard deviation (SD) of the protein abundance level across different samples, respectively. The heat maps could visualize the fold changes in protein abundance levels. Next, to verify the statistical significance of the proteins, we performed Student *t* tests for each pair of experimental groups and only retained proteins with *P* values smaller than 0.05. We utilized the R packages pheatmap (pheatmap: Pretty Heatmaps; R package version 1.2, 2012), ggpubr (ggpubr R Package: ggplot2-Based Publication Ready Plots, 2020), and ggplot2 to visualize the heat maps.

The protein pathways were analyzed based on the Kyoto Encyclopedia of Genes and Genomes (KEGG) database (https://www.genome.jp/kegg/). The KEGG pathways for each protein were organized, and pathways related to angiogenesis and immunomodulation were selected to identify the most overlapping pathways using Python code. Subsequently, the proportion (rich factor) and number of proteins included in each pathway were calculated using the total proteins and our proteins related to the pathway. To address the issue of numerous false positives due to the large number of comparisons, even if the *P* value was small, the Benjamini–Hochberg correction was applied to express the results as adjusted *P* values (*P*_adj_).

### Statistical analysis

The results are presented as mean ± SD. Statistical analyses were conducted using one-way analysis of variance with Tukey’s post hoc tests. The number of iterations for each experiment is listed in the corresponding captions. Statistical significance was set at *P < 0.05 and **P < 0.001.

## Results

### Tuning the mechanical properties of the culture matrix using GelMA hydrogel

**Fig. 2.**
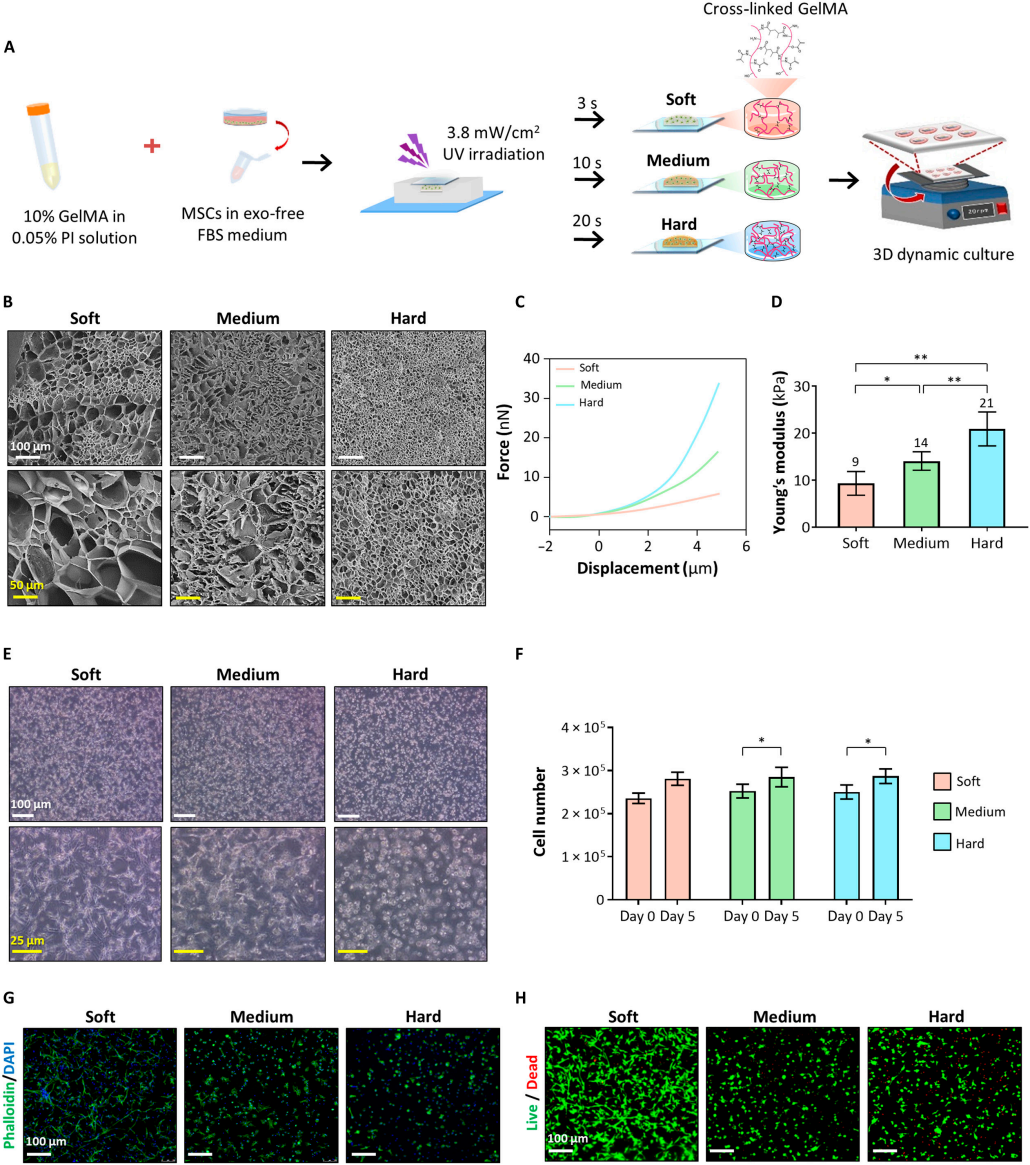
Evaluation of our established bioprocess for modulating cell behavior through mechanical property adjustments using the GelMA hydrogel. (A) Schematic experimental flow of our established bioprocess. MSCs were encapsulated in cross-linked GelMA with different mechanical stiffnesses, namely, soft (3.8 mW/cm^2^ for 3 s), medium (3.8 mW/cm^2^ for 10 s), and hard (3.8 mW/cm^2^ for 20 s), regulated using ultraviolet (UV) radiation. (B) Focused ion beam (FIB) Quanta 3-dimensional (3D) imaging of GelMA hydrogels with different mechanical properties. Scale bars indicate 100 μm (white) and 50 μm (yellow). (C) Force–distance curve of GelMA samples and (D) Young’s modulus values from the force–distance curve obtained using atomic force microscopy (AFM) (*n* = 3). (E) Optical microscopic images of each group of GelMA-encapsulated MSCs after 5 d of culture. Scale bars indicate 100 μm (white) and 25 μm (yellow). (F) DNA quantification of MSCs cultured under each cross-linked condition after 5 d (*n* = 3). (G) Phalloidin staining images of encapsulated MSCs. Phalloidin (green), 4′,6-diamidino-2-phenylindole (DAPI, blue). Scale bar indicates 100 μm. (H) Live/dead images of encapsulated MSCs. Scale bar indicates 100 μm. Statistical analysis was conducted using one-way analysis of variance (ANOVA). *P < 0.05 and **P < 0.01. PI, photoinitiator; FBS, fetal bovine serum.

The mechanical properties of the culture matrix used for 3D MSC culture were adjusted by controlling the UV irradiation time during cross-linking in GelMA hydrogels. We opted for 3 distinct levels of culture matrix mechanical properties, to reflect 3 distinct in situ tissues in our body: soft tissues (soft, less than 10 kPa), muscle (medium, 10 to 15 kPa), and unmineralized bone matrix tissues (hard, 20 to 40 kPa) (Fig. 2A) [15,16]. The culture matrices exhibited different interconnected pore structures and sizes according to the UV exposure time (Fig. 2B). These structural differences in the porous networks led to significantly different mechanical properties in the 3 groups of culture matrices, as measured by atomic force microscopy, i.e., the soft group (9 kPa), the medium group (14 kPa), and the hard group (21 kPa) (Fig. 2C and D). MSCs encapsulated in the GelMA hydrogels were cultured for 5 d in a CO_2_ incubator under dynamic conditions with 3D orbital shaking at 20 rpm, during which the cells grew and exhibited different cell morphologies depending on the mechanical properties of the culture matrices (Fig. 2E and F). As the matrix stiffness decreased, the encapsulated MSCs spread and branched out into the hydrogel. Conversely, MSCs cultured in the stiffer matrices tended to display a rounded shape without spreading branches and were likely physically confined within the hydrogel. The differences in cell morphology were consistent with the varying patterns of actin filament polymerization, with traces of cell locomotion (Fig. 2G). Cells in all groups were highly viable during our 3D culture procedures as barely any dead cells were observed (Fig. 2H).

### Gene expression profiles of MSCs in hydrogels with different mechanical properties

**Fig. 3.**
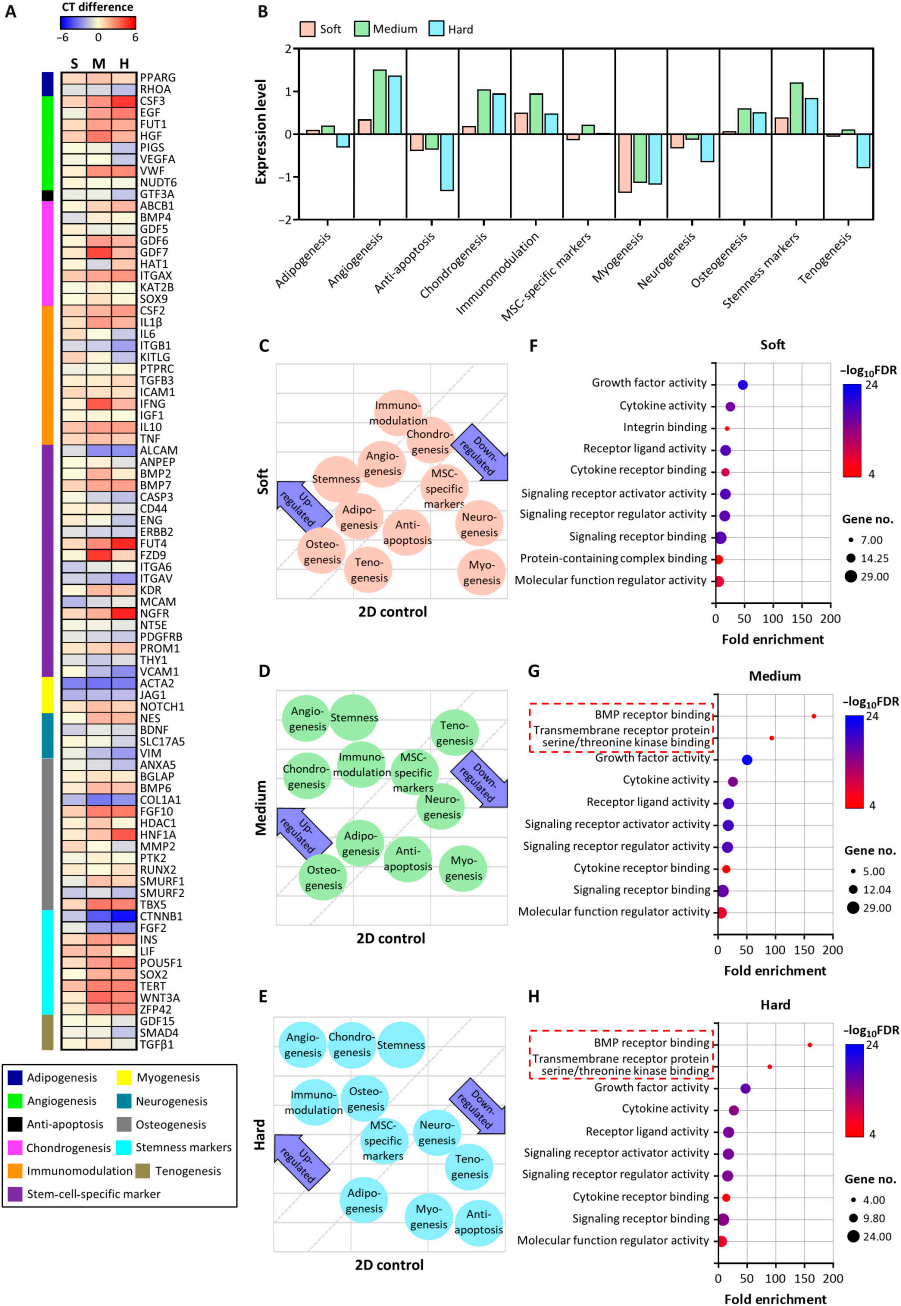
Gene expression profiles of encapsulated MSCs. (A) Heat map of messenger RNA ( mRNA) obtained from 3D-cultured MSCs in the soft (S), medium (M), and hard (H) hydrogels using a quantitative polymerase chain reaction (qPCR) array, compared to the difference in cycle threshold (CT) values with 2D-cultured MSCs (*n* = 3). (B) The organization of gene expression patterns is categorized based on their depth-related associations across each hydrogel group. Scatter plot illustrating the differences in gene expression patterns of the (C) soft, (D) medium, and (E) hard groups compared to that of 2D-cultured MSCs (2D control). Gene Ontology enrichment analysis of MSCs cultured in (F) soft, (G) medium, and (H) hard hydrogels related to the molecular function pathway. FDR, false discovery rate; CSF-2, colony-stimulating factor 2; BMP, bone morphogenetic protein.

We investigated the overall gene expression profiles of MSCs 3D-cultured in different mechanical microenvironments and compared them with those of MSCs cultured on conventional culture plates (2D control), using a quantitative polymerase chain reaction (qPCR) array kit capable of examining 84 key genes reflecting the generic nature of human MSCs. The heat map data of the gene expression profiles of the soft, medium, and hard groups were presented and analyzed after normalization with the 2D control group (Fig. 3A and B). The differential cellular morphologies resulting from varying culture matrix stiffness levels led to distinct gene expression profiles in the MSCs. In particular, the gene expression profile of the soft group appeared to be distinct from those of the other groups, while the medium and hard groups displayed relatively similar gene expression patterns (Fig. 3C to E). Overall, 3D-cultured MSCs exhibited enhanced gene expression associated with angiogenesis, chondrogenesis, immunomodulation, and stemness while expressing levels of MSC-specific markers comparable to those of the 2D control group. The expression of genes related to the immunomodulatory and angiogenic capacities of MSCs, such as epidermal growth factor (EGF), hepatocyte growth factor (HGF), VEGF-A, colony-stimulating factor 2 (CSF-2), and transforming growth factor beta 1 (TGFβ1), was promoted in the medium and hard groups compared to that in the 2D control group [17]. We conducted GO analysis focusing on molecular function pathways by selecting factors that were more abundantly secreted in each hydrogel group compared to the 2D control group (Fig. 3F to H). Specific GO terms were examined based on their fold enrichment, false discovery rate, and the number of genes. The soft group demonstrated superior levels of growth factor activity-related components compared to the 2D control group, attributed to the overexpression of growth factors such as EGF and HGF (Fig. 3F). Notably, the gene expression patterns of the medium and hard groups tended to be associated with pathways related to “bone morphogenetic protein (BMP) receptor binding” and “transmembrane receptor protein serine/threonine kinase (RSK) binding” (Fig. 3G and H). These pathways contribute to signaling pathways related to the phosphoinositide 3-kinase–protein kinase B (PI3K–Akt) pathway, as well as TGFβ and BMP signaling, playing a crucial role in osteogenesis, chondrogenesis, angiogenesis, and immunomodulation [18]. Additionally, these groups exhibit higher levels of components that regulate “cytokine activity”, which plays a significant role in the paracrine effects of MSCs, and factors contributing to “receptor ligand activity”. These results suggest that modulating the mechanical microenvironment of MSCs influences multiple pathways related to angiogenesis and immunomodulatory capacity while maintaining the stemness of stem cells. Therefore, we anticipated that this promotion of the therapeutic capacities of MSCs resulting from differences in their mechanical microenvironment could also modulate their EVs’ therapeutic capacities.

### Characterization of EVs derived from 3D- and 2D-cultured MSCs

**Fig. 4.**
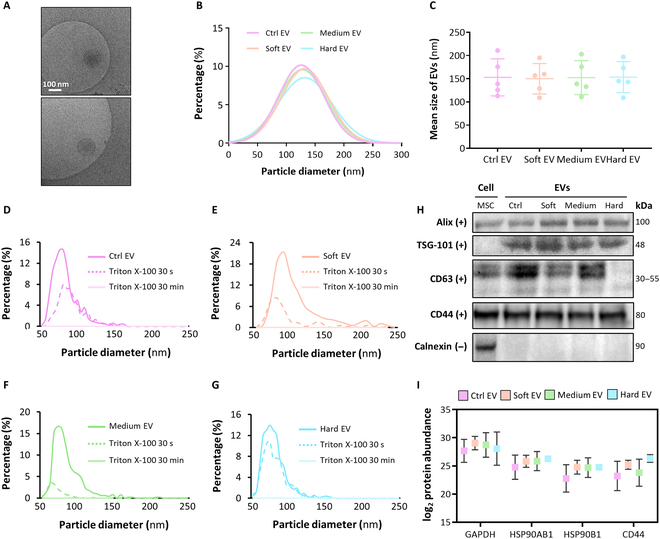
Characterization of MSC-derived EVs collected in this study. (A) Cryo-transmission electron microscopy (cryo-TEM) images of the ctrl EV. The scale bar indicates 100 nm. (B) Graph of size distribution as a number-based percentage and (C) mean size of EVs (*n* = 5). Concentration of EVs and EVs treated with 3% Triton X-100 in (D) ctrl, (E) soft, (F) medium, and (G) hard for 30 s or 30 min. (H) Western blot analysis of EV markers Alix, TSG-101, and CD63; the MSC surface protein CD44; and the negative marker calnexin. (I) Protein abundance of EV markers and glyceraldehyde-3-phosphate dehydrogenase (GAPDH) across each group (*n* = 3). Ctrl EV, 2D control-derived EVs.

The morphologies of the EVs isolated in our study were visually characterized by multiple transmission electron microscopy images, which displayed rounded and vesicular structures of approximately 100 to 150 nm (Fig. 4A). The sizes of the EVs collected from all groups ranged from 30 to 200 nm, similar to the EV sizes reported in many previous studies (Fig. 4B and C). When treated with 3% Triton X-100, which is commonly used for liposomal digestion, all EVs were shown to disappear with time in the size distribution range in which normal EVs were typically detected (Fig. 4D to G). These results verify that the collected EVs were true vesicles enveloped by a lipid bilayer [6]. Western blot analysis was performed using MSCs and EVs from each group to examine the EV markers Alix, TSG-101, and CD63; the MSC surface protein CD44; and the endoplasmic reticulum marker calnexin (Fig. 4H). All groups of EVs were positive for Alix, TSG-101, and CD63, along with CD44, a multifunctional cell surface adhesion receptor highly expressed in the parent cells that is known to be shared with EVs derived from MSCs. The negative marker calnexin was not detected in any of the EV samples. Furthermore, proteomic analysis confirmed the expression of EV markers such as heat shock protein 90 alpha family class beta member 1 (HSP90AB1), HSP90B1, and CD44 (Fig. 4I) [19]. The expression level of glyceraldehyde-3-phosphate dehydrogenase was presented along with the data to show the degree of abundance of EV protein marker expression [20].

### Immunomodulatory and angiogenic efficacy of EVs secreted from 3D- and 2D-cultured MSCs

**Fig. 5.**
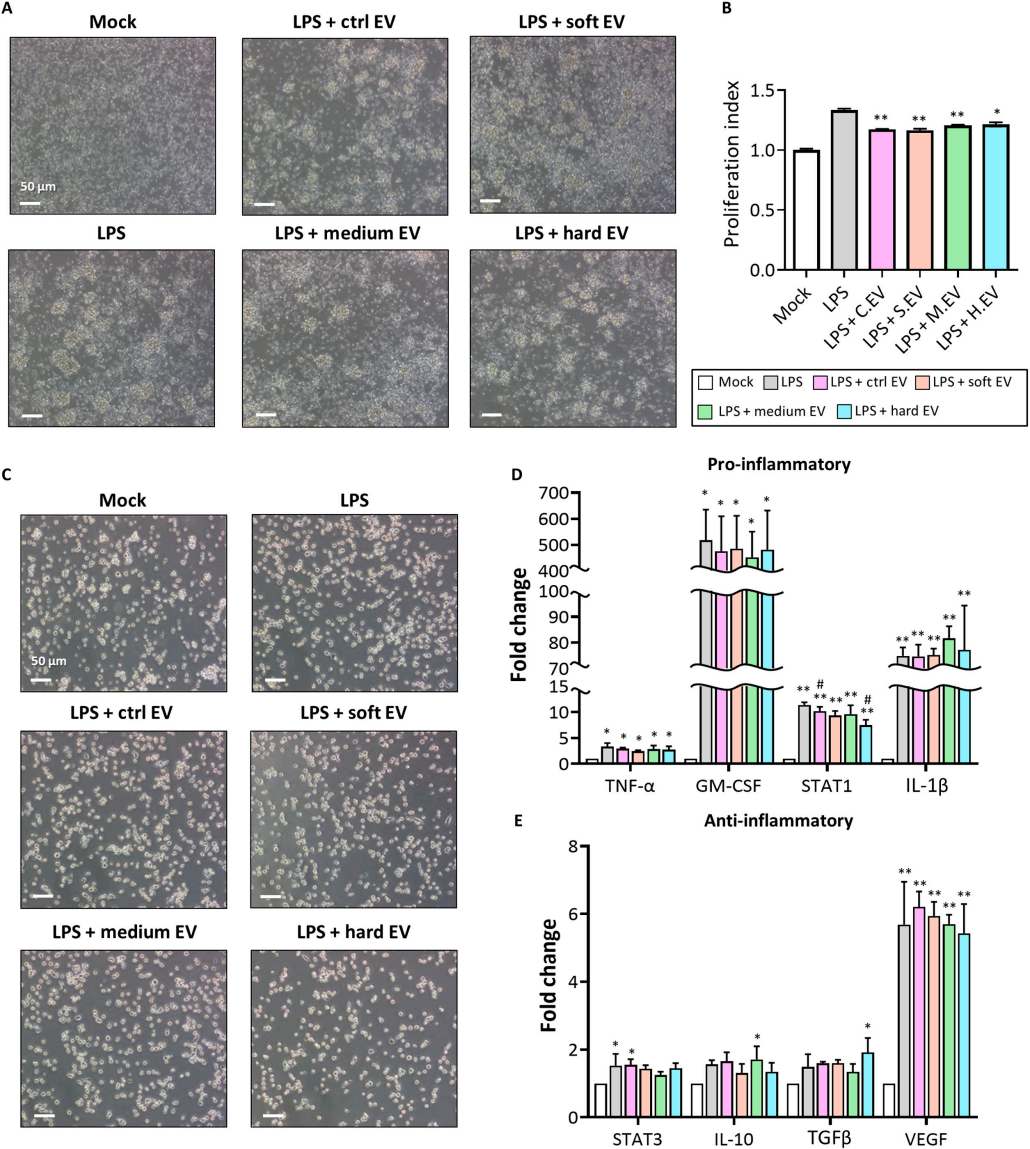
In vitro test for evaluating the immunomodulatory efficacy of the ctrl EV, soft EV, medium EV, and hard EV groups. (A) Optical microscopic images of peripheral blood mononuclear cells (PBMCs) treated with LPS and EVs obtained from each group and (B) quantification of the PBMC proliferation profiles of each treated group compared to that of the negative control (mock) (*n* = 4). The scale bar indicates 50 μm. (C) Optical microscopic images of THP-1 cells treated with LPS and EVs obtained from each group. The scale bar indicates 50 μm. Gene expression related to (D) pro-inflammatory and (E) anti-inflammatory responses using quantitative real-time polymerase chain reaction reverse transcription (qRT-PCR) (*n* = 3). Data are presented as mean ± SD; *P < 0.05 and **P < 0.01 compared to the mock group and ^#^*P* < 0.05 compared to the LPS group. Statistical analysis was performed using one-way ANOVA. TNF-α, tumor necrosis factor-α; GM-CSF, granulocyte-macrophage colony-stimulating factor; IL-1β, interleukin 1 beta.

We evaluated the immunomodulatory efficacy of EVs obtained from MSCs cultured in varying 3D mechanical microenvironments. This was done by co-treating immune cells, including PBMCs and THP-1, with the EVs and LPS. LPS treatment promoted PBMC proliferation and increased the frequency of cell colony agglomerations compared to the negative control group (mock; PBS) (Fig. 5A and B). However, PBMCs co-treated with LPS and EVs showed significant decreases in the LPS-induced proliferation of PBMCs, while no significant differences were found among the EV-treated groups (Fig. 5B). In addition, LPS treatments triggered the M1 polarization of THP-1 cells, showing significantly increased gene expression levels of tumor necrosis factor-α, granulocyte-macrophage colony-stimulating factor (GM-CSF), signal transducer and activator of transcription 1 (STAT1), and interleukin 1 beta (IL-1β) while displaying cell morphology similar to that of the mock group (Fig. 5C and D). Interestingly, the hard EVs appeared to inhibit the LPS-induced M1 polarization of THP-1 cells, as the STAT1 gene expression was down-regulated in the hard group compared with that in the LPS-only group. Regarding the macrophage phenotype 2 (M2) polarization of THP-1 cells, the LPS-treated groups tended to show increased gene expressions of STAT3, IL-10, TGFβ, and VEGF, but the degrees of increase (fold changes) were found to be far smaller (insignificant in most groups) than those shown in the gene expression changes related to M1 polarization (Fig. 5E). Interestingly, IL-10 and TGFβ gene expression levels were up-regulated in the medium and hard EVs, respectively, compared to those in the mock, which could suggest that the EVs collected from the medium and hard EVs contribute to the M2 polarization of THP-1 cells.

**Fig. 6.**
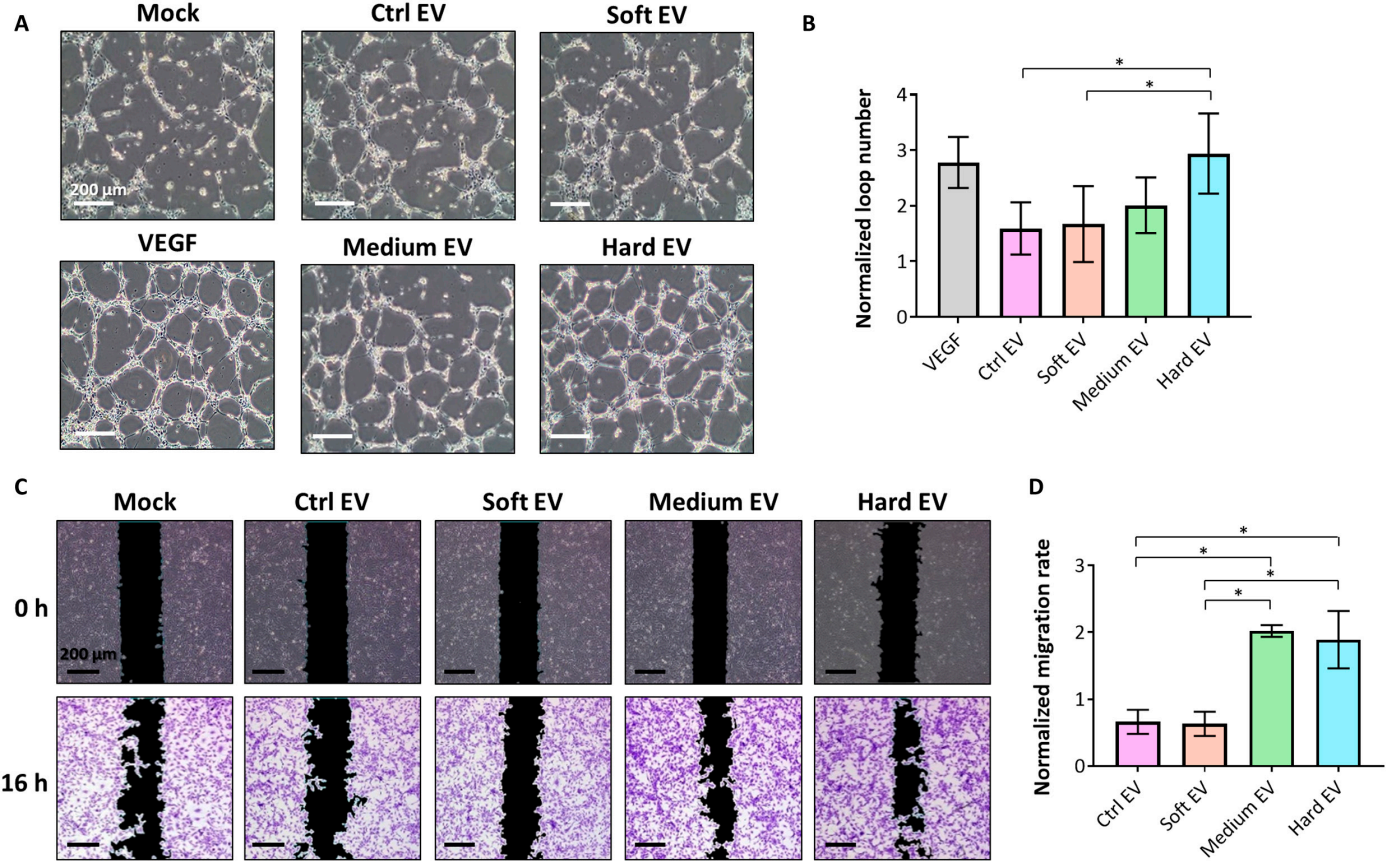
In vitro test for evaluating the angiogenic efficacy of the ctrl EV, soft EV, medium EV, and hard EV groups. The angiogenic efficacy of each group of EVs was evaluated by (A) HUVEC tube-formation assay and analyzed by (B) the number of loops normalized to the mock group (*n* = 4). The scale bar indicates 200 μm. The angiogenic efficacy of the EVs was also investigated by (C) the HUVEC wound-healing assay and analyzed by (D) the migration rate normalized to the mock group (*n* = 3). The scale bar indicates 200 μm. Statistical analysis was performed using one-way ANOVA. *P < 0.05 and **P < 0.01.

In terms of the angiogenic capacity of the EVs, the tube-formation rate of HUVECs was significantly promoted in the hard group compared to those in the 2D control-derived EV (ctrl EV) and the soft group (soft EV), showing a result comparable to that of the positive control group treated with VEGF (Fig. 6A and B). Likewise, the results of our wound-healing assay revealed that HUVEC migration rates were significantly increased by the EVs collected from 3D-cultured MSCs with harder matrices, such as those of the medium and hard groups (medium EVs and hard EVs; Fig. 6C and D).

### EV cytokine cargos according to different culture matrix properties

**Fig. 7.**
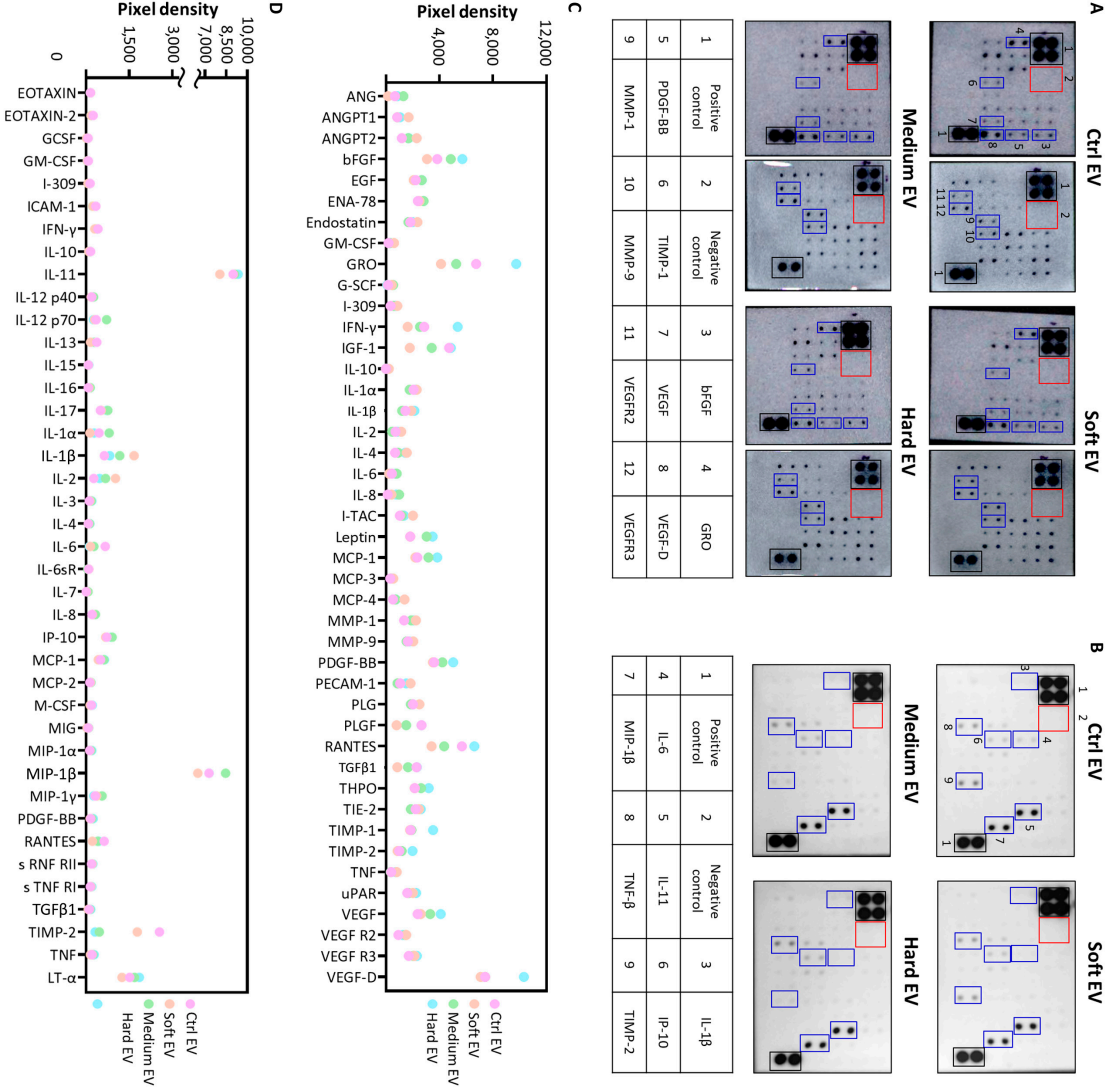
Evaluation of cargo contents in encapsulated MSC-derived EVs compared to 2D control-derived EVs (ctrl EV), using a cytokine array. Spot images of cytokine arrays associated with the (A) angiogenesis and (B) immunomodulation of the EVs from each group. Quantification of the cytokine profiles of the EVs obtained from each group related to (C) angiogenesis and (D) immunomodulation functions. GRO, growth-regulated oncogene; PDGF-BB, platelet-derived growth factor-BB; TIMP-1, tissue inhibitor of metalloproteinases 1; MMP-1, matrix metalloproteinase-1; MMP-9, matrix metalloproteinase-9; FGF, human fibroblast growth factor-basic; IP-10, interferon gamma-induced protein 10.

Various cytokines present in the EVs, related to angiogenesis and inflammation, were analyzed using commercial cytokine array kits (Fig. 7A and B). The expression levels of cytokines in the EVs were detected as spots in the array, and their levels were presented as the pixel densities of the spots, which were quantitatively plotted after normalization with the positive control, according to the manufacturer’s instructions; they were compared among all 3D-cultured groups (soft, medium, and hard EVs) and the 2D-cultured group (ctrl EV) (Fig. 7C and D). As expected, EVs from all MSC groups contained various therapeutic cytokines related to angiogenesis and immunomodulation but the expression levels appeared to differ among the groups to a certain degree. For example, the medium and hard EVs tended to express higher levels of pro-angiogenic cytokines than the other groups, particularly bFGF, growth-regulated oncogene (GRO), platelet-derived growth factor-BB (PDGF-BB), tissue inhibitor of metalloproteinases 1 (TIMP-1), VEGF, and VEGF-D. Unlike the angiogenic cytokine inclusions in the EVs, no differentiated expression trends depending on the culture matrix properties were observed in the immunomodulatory cytokines.

### Overall protein expression profiles in the EVs analyzed by LC–MS and bioinformatics

**Fig. 8.**
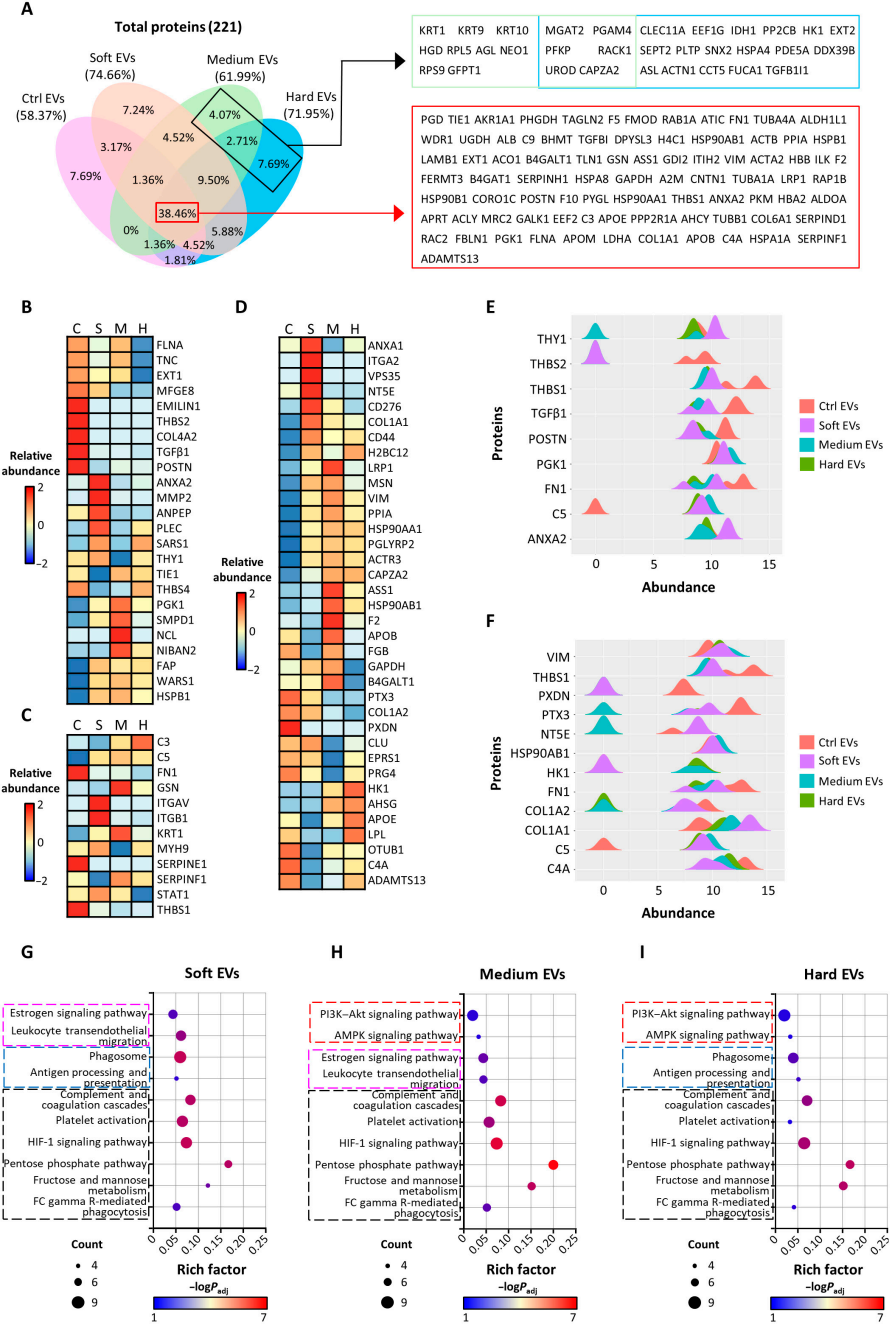
Proteomic profiling of EVs was conducted through liquid chromatography–mass spectrometry (LC–MS) (*n* = 3). (A) Venn diagram depicting the proteins secreted from each group of EVs, including lists of the unique proteins in the medium and hard groups and proteins secreted in all groups. Heat map diagrams of proteins related to (B) angiogenesis, (C) both angiogenesis and immunomodulation, and (D) immunomodulation, analyzed through relative abundance. Ctrl EVs are represented as C, soft EVs as S, medium EVs as M, and hard EVs as H. Comparison of significant (*P* < 0.05) genes related to (E) angiogenesis and (F) immunomodulation presented by a wave figure. Kyoto Encyclopedia of Genes and Genomes (KEGG) pathway enrichment analysis of (G) soft EVs, (H) medium EVs, and (I) hard EVs compared to ctrl EVs. PTX3, pentraxin 3; POSTN, periostin; VIM, vimentin; C5, complement component 5; PXDN, peroxidasin; PI3K–Akt, phosphoinositide 3-kinase–protein kinase B; AMPK, AMP-activated protein kinase; HIF-1, hypoxia-inducible factor-1.

The LC–MS analysis provided the overall quantitative protein expression profiles of the EVs collected from all 3D-cultured and 2D-cultured groups. In total, 221 proteins were detected across all groups, and the proportions of common and distinct expressions among the groups are presented in a Venn diagram (Fig. 8A). A total of 129 proteins were identified in the ctrl EVs (58.37%), whereas the soft, medium, and hard EVs contained 165 (74.66%), 137 (61.99%), and 159 proteins (71.95%), respectively. EVs from the 3D-cultured groups exhibited greater protein diversity than those from the 2D control. Notably, hard EVs, which exhibited higher efficacies in immunomodulation and angiogenesis, revealed the presence of 17 unique proteins (7.69%), including C-type lectin domain family 11-member A (CLEC11A) and TGFβ1-induced transcript 1 (TGFβ1I1), whereas all groups contained several factors related to angiogenesis and immunomodulation, such as TGFβ1, fibronectin 1 (FN1), and pentraxin 3 [21,22].

To compare the differentiated effects of MSC mechanical microenvironments on EVs, we initially refined the extensive protein expression profile dataset through bioinformatics and then used a Web-based big data platform for GO and annotation (EBI QuickGO) and a text-matching algorithm to identify proteins relevant to angiogenesis and immunomodulation. The 221 proteins were selectively presented as angiogenesis-related, both angiogenesis- and immunomodulation-related, or immunomodulation-related proteins using heat map diagrams (Fig. 8B to D). Overall, all groups exhibited highly up-regulated expression levels of proteins related to angiogenesis and immunomodulation, while the expression patterns varied among the groups. For immunomodulation, a tendency toward more pronounced protein expression patterns was observed in the 3D-cultured groups (Fig. 8D). Proteins with significant differences (*P* < 0.05) in abundance levels compared to other groups were separately visualized as wave plots based on the absolute abundance levels of each protein (Fig. 8E and F). The results showed that proteins that play significant roles in angiogenesis and immunomodulation were highly expressed in all groups. All EV groups contained TGFβ1, periostin (POSTN), and FN1, which are related to the up-regulation of VEGF, and thrombospondin-1 (THBS1), known to inhibit the pro-angiogenic actions of FGF-2, and expressed MSC-EV markers such as HSP90AB1 and vimentin (VIM) [23]. Notably, THBS2, known as a potent inhibitor of angiogenesis mediated by the THBS2 receptor CD36, was expressed at significantly high levels in the ctrl EVs (Fig. 8E). The ctrl EVs also showed up-regulation of peroxidasin, which aids in angiogenesis (Fig. 8F) [24].

To further investigate the pathways in which the EV proteins are involved, we performed KEGG enrichment analysis. The KEGG results reveal various pathways of all proteins, including those related to cell biogenesis; however, these results do not sufficiently explain the differences in therapeutic efficacy among the group’s EVs. In order to better understand the significant differences in therapeutic efficacy, we removed the overlapping pathways between the ctrl EV and hydrogel group KEGG datasets. We then presented the top 10 pathways related to angiogenesis and immunomodulation, based on the number of proteins and the rich factor (Fig. 8G to I). All 3D-cultured groups showed greater contributions in 6 pathways, including “complement and coagulation cascades”, than the ctrl EVs (marked as black in the figure). Notably, the secretion of pentose phosphate, which can activate the secretion of miR-93, known to reduce oxidative stress and promote angiogenesis, was the highest in the medium EVs [25]. Additionally, the medium and hard EVs showed significant enrichment in “fructose and mannose metabolism” compared to the ctrl EVs. Mannose induces regulatory T cells and suppresses effector T cells, thereby enhancing immunomodulation [26]. Fructose metabolism activates AMP-activated protein kinase (AMPK) signaling and mitochondrial respiration, promoting angiogenesis, a characteristic frequently observed in cancer cells and tumor growth [27]. Accordingly, the medium and hard EVs showed significant contributions to the PI3K–Akt and AMPK pathways compared to the soft group (red). The “estrogen signaling pathway”, which is known to regulate the nuclear factor kappa B and interferon pathways, and the “leukocyte transendothelial migration” pathway were identified in the soft and medium EVs (pink). Additionally, the “phagosome” and “antigen processing and presentation” pathways were identified in the soft and hard EVs (blue). In summary, the 3D-cultured groups tended to contribute more to angiogenesis and immunomodulation than the ctrl EVs, with the medium and hard EVs showing greater potential than the soft EVs.

### MicroRNA expressions according to different culture matrix properties

**Fig. 9.**
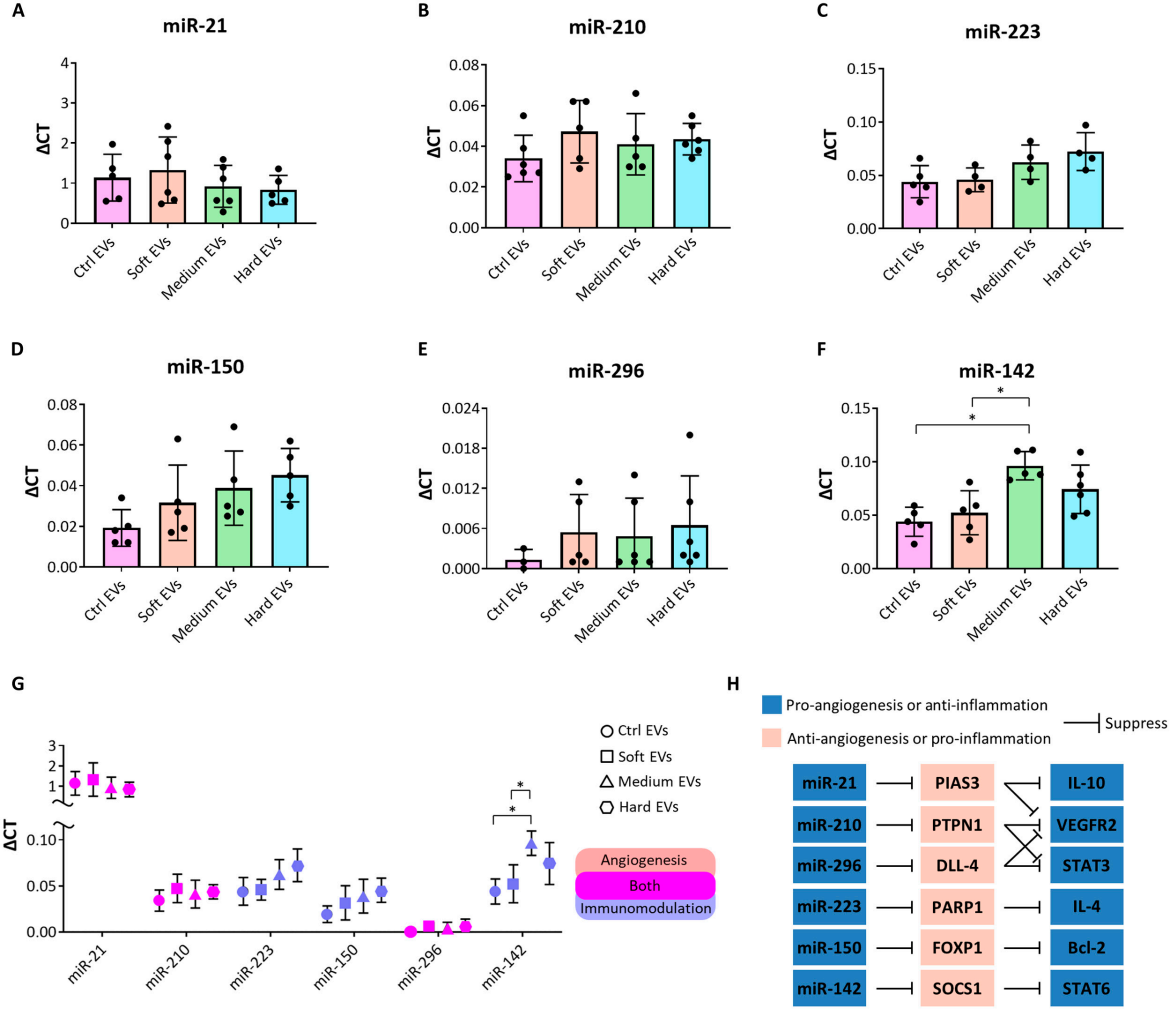
Comparison of the microRNA (miRNA) contents in the EVs from each group. Analysis of (A) miR-21, (B) miR-210, (C) miR-223, (D) miR-150, (E) miR-296, and (F) miR-142, which are associated with angiogenesis and immunomodulation, normalized by miR-16 (*n* = 3 to 6). (G) Comparison of the delta CT (ΔCT) values of miRNAs secreted by each group and (H) miRNA pathways contributing to angiogenesis and immunomodulation. Statistical analysis was conducted using one-way analysis of variance. *P < 0.05. PIAS3, protein inhibitor of activated STAT3; PTPN1, phosphatase nonreceptor type 1; DLL-4, delta-like ligand 4; PARP1, poly(ADP-ribose) polymerase 1; FOXP1, forkhead box protein P1; SOCS1, suppressor of cytokine signaling 1.

We also investigated the contents of miRs in the EVs using qRT-PCR (Fig. 9). The expression levels of miR-21, miR-210, and miR-296, known to facilitate T helper 2 cell and M2 polarization and angiogenesis, and miR-223, miR-150, and miR-142, which are involved in immunomodulation, were high in all groups without significant differences (Fig. 9A to F) [28,29]. These miRs, apart from miR-296, were sufficiently expressed in all EV groups with the highest miR-21 expression, and in particular, miR-142 was significantly overexpressed in medium EVs (Fig. 9G). The evaluated expression of the individual miRs suggests the potential to suppress pro-inflammatory-related factors, such as protein inhibitor of activated STAT3 and protein tyrosine phosphatase nonreceptor type 1 (PTPN1), thus activating anti-inflammatory and pro-angiogenic factors such as IL-10, STAT3, and IL-4 (Fig. 9H) [30]. Notably, significantly increased expression of miR-142 in the medium EV group could result in the inhibition of the suppressor of cytokine signaling 1 (SOCS1), inducing the expression of STAT6, and thereby promoting immunomodulation [31].

## Discussion

The ECM provides structural support around cells and plays a crucial role in various physiological functions, including cell survival, growth, movement, and differentiation [32]. Each cell type requires a different favorable microenvironment, where cells interact with the ECM and communicate through integrins and Piezo1, a type of transmembrane protein, which significantly influences cellular behaviors [33]. Therefore, the manipulation of the various physical and biochemical properties of hydrogels has been widely reported [34]. GelMA is considered a potentially attractive hydrogel material to represent ECM microenvironments for culturing adhesive cells [32]. The mechanical properties of the GelMA hydrogel can be readily controlled by adjusting the conditions of hydrogel cross-linking, such as the concentration of the GelMA hydrogel solution and the UV light intensity and exposure time [35]. In this study, MSCs were exposed to controlled 3D-physical interactions with their surrounding culture matrices using GelMA hydrogel. The UV light exposure time was serially adjusted to obtain well-distinguished mechanical properties among the culture groups, namely, the soft (~9 kPa), medium (~14 kPa), and hard groups (~21 kPa). FIB Quanta 3D images revealed correspondingly increasing pore sizes in the culture matrices alongside decreasing mechanical properties, presenting successfully differentiated MSC phenotypes and cytoskeleton polymerization. MSCs can be influenced by the surrounding microenvironment, leading to changes in cell behavior that affect characteristics such as cell proliferation and differentiation capacity and lead to variations in secreted factors [36]. Particularly, in 3D microenvironments with relatively high matrix stiffness, the expression of factors involved in angiogenesis, such as EGF, HGF, and VEGF, increased, and the expression of MSC markers was higher than that in the 2D control. According to previous research, modulating the stiffness of the ECM not only increases the expression of VEGF but also enhances the internalization of VEGF receptor 2, suggesting the potential for greater angiogenesis and immunomodulation efficacy [37]. Furthermore, many factors involved in the BMP pathway, which is known to enhance the Smad pathway, were identified, thereby promoting the expression of key factors in angiogenesis and immunomodulation, such as VEGF, PDGF, and TGF [38]. The transmembrane proteins, such as Piezo1, might be regulated in the medium and hard groups due to the relatively high mechanical stiffness of the microenvironments, which can stimulate the RSK-related pathway. Activation of the RSK-related pathway, contributing to TGFβ superfamily receptor activation, suggests that culturing MSCs in microenvironments with medium and hard levels of stiffness leads to increased expression of multiple factors related to angiogenesis and immunomodulation and, consequently, their EVs are expected to carry higher amounts of these therapeutic factors [39]. EVs derived from MSCs have shown therapeutic efficacy in anti-inflammation and pro-angiogenesis, similar to their parent cells. Our EVs, which are expected to carry therapeutic factors related to angiogenesis and immunomodulation, inhibited the proliferation of LPS-induced blood cells and exhibited anti-inflammatory efficacy by inhibiting M1 polarization and promoting M2 polarization. In particular, the medium and hard EVs demonstrated not only immunomodulatory capabilities but also pro-angiogenic efficacy comparable to that of VEGF, as evidenced by their ability to promote tube formation and migration. The results highlight the potential of modulating the therapeutic efficacy by controlling the mechanical microenvironment surrounding MSCs, underscoring the regulatory effects on the therapeutic efficacy of MSC-EVs as well. According to the MSC qPCR array results, the medium and hard groups expressed diverse genes related to anti-inflammatory and pro-angiogenesis processes, suggesting that the secreted EVs exhibit efficacy through complex mechanisms involving these factors [40]. As expected, pro-angiogenic cytokines, including bFGF, GRO, PDGF-BB, TIMP-1, VEGF, and VEGF-D, showed higher expression in the medium and hard groups, encompassing the secretion of various cytokines across all groups. The results indicate that MSCs cultured in relatively stiffer microenvironments showed up-regulated gene expression associated with angiogenesis and immunomodulation and confirm the expression of various pro-angiogenic cytokines in MSC-EVs. This cytokine profile regulates angiogenesis, and while direct differences in anti-inflammatory cytokines were not observed, it suggests immunomodulatory functions through complex cytokine interactions.

To further investigate the changes in EV cargo, the LC–MS analysis was conducted along with bioinformatic analysis. As different microenvironment conditions can alter the gene expression levels in MSCs and subsequent cytokine secretion in EVs, variations in matrix properties also lead to differences in the protein cargo of EVs. The 3D hydrogel culture induced changes in VEGF and other factors in MSCs and their EVs, aligning with the observation that proteins such as CLEC11A and TGFβ1I1—known for their crucial roles in vascular regeneration through VEGF—were expressed only in hard EVs. Conversely, THBS2, which inhibits the VEGF pathway, was notably present in the control group, correlating with lower VEGF expression in the MSCs compared to other groups. All of these expression differences occur without variation in HSP90AB1, an EV marker, and VIM, an MSC-EV marker. In the angiogenic and immunomodulatory efficacy tests, the medium and hard groups exhibited higher efficacies despite subtle differences in the secretion profiles of key factors related to angiogenesis and immunomodulation among the groups. The therapeutic efficacy of angiogenesis and immunomodulation is regulated by complicated correlations among various factors [41]. Correspondingly, the medium and hard EVs showed up-regulated PI3K–Akt and AMPK pathways, as well as up-regulated “pentose phosphate pathway” and “fructose and mannose metabolism” compared to the soft EVs. When transmembrane proteins such as Piezo1 on MSCs are exposed to high mechanical properties, they contribute to signaling pathways that stimulate the PI3K–Akt and AMPK pathways [42]. In line with this, EVs secreted by the stimulated MSCs (in the medium and hard groups) were found to naturally contain proteins related to the PI3K–Akt and AMPK pathways. The enhanced PI3K–Akt and AMPK pathways also involve the hypoxia-inducible factor-1 signaling pathway, ultimately supporting VEGF expression [43,44]. Additionally, fructose metabolism activates AMPK signaling, so the 2 pathways uniquely observed under medium and hard culture conditions can collectively explain the superior therapeutic efficacy compared to that under soft group conditions. These results suggest that the mechanical properties of the medium and hard conditions (probably above the medium group’s properties in our conditions) might stimulate Piezo1, leading to the up-regulation of related genes and, consequently, an increased presence of PI3K–Akt and AMPK pathway-associated proteins in EVs. We believe that the development of cell regulation through mechanosensing threshold, which can control specific pathways, will enable more precise and detailed modulation of EV-derived therapeutic factors. MiRs are also known as key therapeutic factors in EVs, regulating protein expression directly or indirectly through posttranscriptional regulation. MiR-142, known to facilitate the up-regulation of the pro-angiogenic transcription factor STAT6, was up-regulated in EVs from the medium group, consistent with the observed increase in angiogenesis efficacy. Known for its role in suppressing THBS secretion, the up-regulated expression of miR-142 in the medium EVs demonstrates the down-regulation of THBS compared to that in the ctrl EVs [45].

The main clinical issues with MSC-derived EV therapies can be recognized as a series of important challenges: compliance with current legal requirements regarding the design of large-scale manufacturing processes applicable for “good manufacturing practice”, the improvement of therapeutic efficacy in line with quality control and safety, and the maintenance of the naïve characteristics of the parent MSCs [46]. Previously, conventional 2D culture methods were found to deteriorate MSC functionalities associated with replication, colony-forming ability, and differentiation potential, while the expression of genes related to stemness were significantly down-regulated over culture time [47]. In this study, we present a novel approach for advancing the clinical applications of EV therapies by modulating the cellular microenvironment without any genetic modification or additional biochemicals, maintaining high levels of gene expression associated with specific MSC markers. The mechanical properties used in this study ranged from 5 to 30 kPa to regulate cellular behavior. However, in situ tissues exhibit a much broader and more diverse range of stiffness [48]. Since the properties of GelMA hydrogels can be further adjusted by modifying the degree of methacrylation, future investigations could explore ways to fine-tune the surrounding mechanical microenvironment. This, in turn, may provide greater control over cellular behavior and EV characteristics, expanding the potential applications of this approach. Furthermore, the previously mentioned PI3K–Akt pathway, influenced by mechanosensing, also contributes to the secretion of factors such as osteocalcin and runt-related transcription factor 2, which, in turn, leads to the activation of pathways related to chondrogenesis and osteogenesis [49]. 

While our study primarily focused on evaluating the effects related to angiogenesis and immunomodulation, a more detailed and broader range of hydrogel control could be explored in future studies. Under such conditions, we expect that the secreted EVs will carry factors associated with these additional pathways. Our comprehensive proteomic and miR analysis results indicate that various therapeutic factors in EVs associated with angiogenic and immunomodulatory efficacy, including those with significant differences and those with only minor variances, are regulated by modulating the mechanical microenvironment surrounding MSCs.

## Data Availability

Data are available from the corresponding author upon reasonable request.
